# Sustainable and economical alternatives to fragment capture materials in explosive and ballistic trials

**DOI:** 10.1007/s12024-024-00797-5

**Published:** 2024-03-12

**Authors:** James Read, Philip Quinlan, Susie Bloodworth-Race, Rachael Hazael, Richard Critchley

**Affiliations:** Cranfield Forensic Institute, Cranfield University, Defence Academy of the United Kingdom, Shrivenham, SN6 8LA UK

**Keywords:** Strawboard, Blast, MDF, Fragmentation, Explosive

## Abstract

Strawboard has been utilised as a fragmentation capture material since the 1960s, mainly employed to capture fragments from explosives and explosive devices from arena trials of munitions. As this material has historically been calibrated to a known standard, it has a proven record of allowing research establishments to ascertain the velocity of a fragment based on the depth of penetration of the strawboard. During the time of calibration, strawboard was used as a common building material which was both widely available and relatively affordable; however, due to the recent economic crisis and geopolitical supply issues, this is no longer the case. Building on initial testing, this paper investigates alternatives to strawboard to determine if a cheaper, more readily available material can be used instead. The alternatives are compared and judged based on the NATO ARSP-03 guideline for capture material which includes metrics such as price and attainability, as well as assessing environmental impact and its ability to be used as a viable alternative to strawboard in an explosive environment. Based on these NATO guidelines, explosive fragmentation and ballistic experiments were conducted, and ten materials were tested based on the following criteria: Handling, Density, Flammability, Calibration, Cost and Availability. Medium Density Fibreboard (MDF) was found to be a suitable alternative to strawboard. The data demonstrates that it provides the same capture performance as strawboard at approximately a quarter of the cost and is far more readily available. Other materials also showed potential and further testing should be undertaken to validate these materials as alternatives to MDF.

## Introduction

Historically, the ability to test fragmentation has resulted in an increased understanding of both the lethality and performance of weapon systems [1–4]. However, a fundamental understanding of the injury to the human body [5] is crucial to developing enhanced protection mechanisms for both personnel [6–8] and equipment [9–11].

Traditionally, analysis of the effects from explosive fragmentation have been undertaken using a series of strawboard panels, where the key metric under examination is Depth of Penetration [12–15]. The only material supplier available to the UK manufactures strawboard from paper which is pressed together to form a rigid structure [16]. Prices for the supply of such material have increased in recent years due to both the manufacture and supply being provided by a single organisation [17]. As reported previously, this is no longer a viable option when both time and cost penalties are becoming an ever more present risk to research programmes, and economical alternatives require quantification [18].

**Fig. 1:**
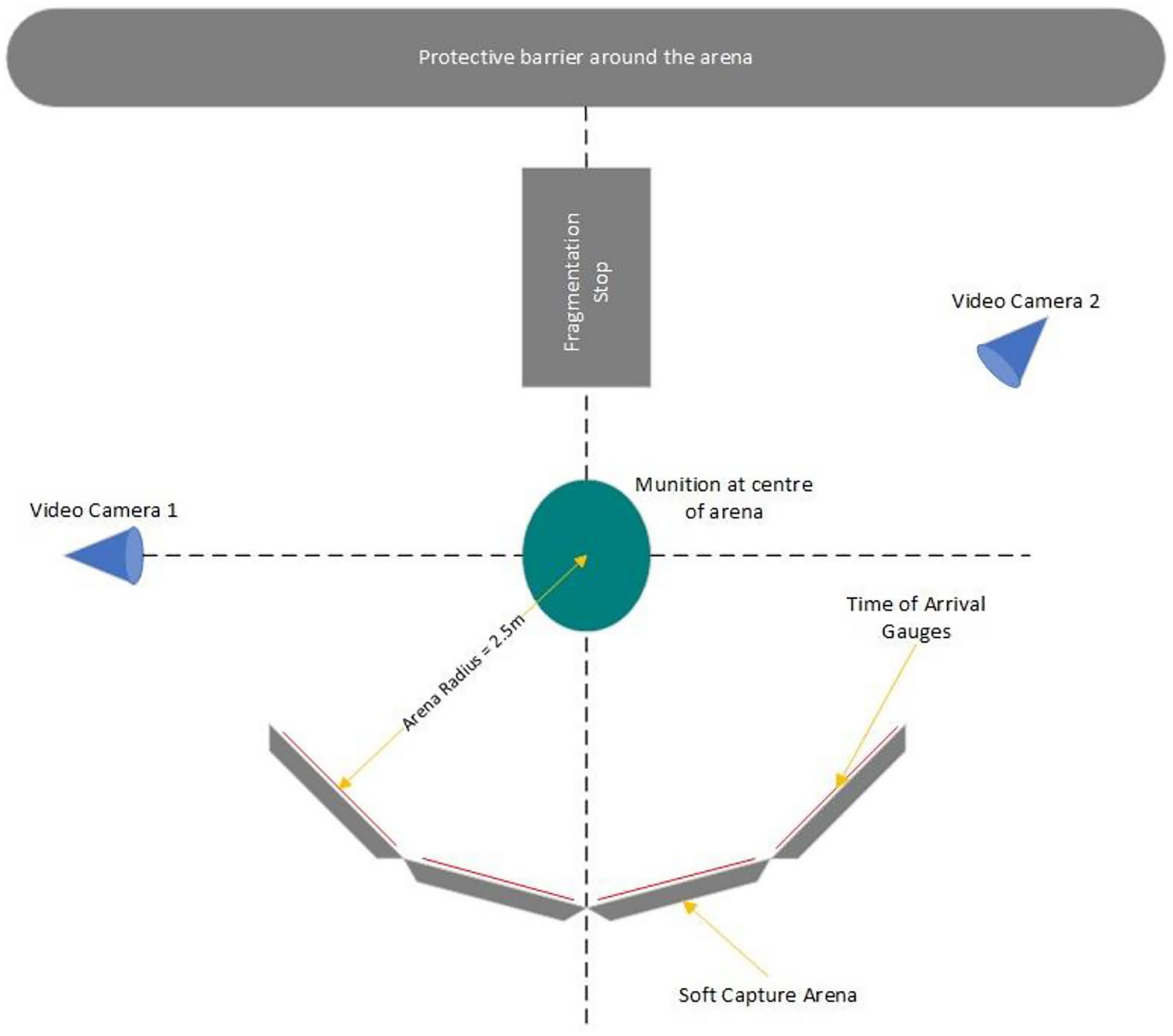
Arena Trial [27]

Recent acts of terrorism [19–21] have highlighted the importance of understanding both the distribution, geometries, and the interaction of fragmentation under varying conditions [22–26]. The most common types of trials for assessment of fragmentation involve creating a semi-circle of witness capture materials that are located around a central point from which the fragmentation will be expelled from an explosive charge [27]. Figure 1 has traditionally used strawboard as their material of choice to record pattern spread and depth of penetration in accordance with ARSP-03 [28], alongside the additions of metallic foil to measure arrival and departure time (velocity) of the fragments between singular or multi-layered panels [25, 29, 30]. In addition, maturation of technology has now allowed researchers to use High Speed Video (HSV) footage to gain a deeper understanding not only of the fragmentation performance but also the blast phenomena [31–33].

When considering the arena trial diagram above, a traditional set up would utilise ~ 1800 sheets of strawboard to cover an area of 15m with a layered configuration of six panels deep and two panels high. At a cost of £12^1^ per strawboard panel (dependant on size and depth), this equates to ~ £21,000 for a single test. When statistical analysis is considered, a minimum of three tests should be repeated and as such the total cost could exceed £63,000 (at the time of writing). This cost is unsustainable and with the UK relying on a single supplier [17], is at risk of escalating costs through supply chain issues.

Whilst the ability to measure arrival / departure times of fragmentation and suitable assessment from HSV are important, any alternative materials must conform to the requirements laid out in NATO publication ARSP-03 [28]:

- Handling – the size and mass of soft capture material should enable two persons to handle it. The material should also retain enough structural integrity to be handled when partially saturated,
- Density – the soft capture material should not affect the physical characteristics of the impacting fragment,
- Flammability – any material used should minimize the risk of fire when containing hot fragments or sufficient precautions should be taken if a flammable material cannot be avoided,
- Calibration – material used should (where possible) be calibrated to give a measure between depth of penetration and impacting speed,
- Cost – the material used should not be excessively expensive as to limit the number of firings,
- Availability – The material shall be readily available in the region of the testing.

Although these are metrics of importance, environmental impact is negated in the standard but must be considered to ensure research establishments are coherent with the UK government ambition to be carbon neutral by 2050 [5, 34]. The preference to avoid costly materials that become a limiting factor in experiments should also be noted.

The review of the literature above has shown that although an initial study within the UK has proposed the use of both Medium Density Fibreboard (MDF) and underlay as materials with viable performance characteristics [18], a more detailed study is required on more materials to explore their feasibility in the field of explosive testing.

The aims of this work are therefore; to expand on the response classification detailed in previous works [18], and enhance the data sets by analysing an additional seven materials in addition to confirming the results shown during the preliminary study [18]. Both ballistic and explosive regimes are investigated to enable direct comparison of material response between both commonly used experimental methods used within reconstruction of crime. The results and subsequent analysis from this work would allow for informed decisions based on scientific data to be made on cost effective alternatives to strawboard with no inconsistencies to data sets, thereby bringing greater value for money to both research and forensic applications.

## Materials and methods

### Materials

Ten target materials were investigated in this study: strawboard, MDF, underlay, plywood, chipboard, bamboo, and hardboard due to their similar weight, size, or construction to strawboard, and polyvinyl chloride (PVC), polycarbonate and Plexiglass/Perspex due to their widespread availability and low cost (Table 1). Previous work using strawboard, MDF, and underlay has shown promise in both lethal and less-than-lethal kinetic scenarios [18], whilst the remaining materials were selected due to cost effectiveness and performance against emergent threats [6] (Table 2).

**Table 1:** Comparison of material thickness

**Material**	**Thickness range (mm)**	**Density (kg/m**^3^**)**	**Precursors**
Strawboard	3.7 mm	2.72	Good quality recycled paper [18]
MDF	4	500–1000	Wood residuals ground in a steam environment and combined with wax and a resin binder.
MDF	6		
MDF	18		
Underlay	4	33,850	Various, including wood chips, plant fibres, softwood flakes, sawdust, paper. Pressed into resin boards as with MDF.
Plywood	8	580–620	Thin sheets of 90 degree laminated wood, helmet together with starch paste or glue.
Chipboard	9	Variable depending on requirements 620–640 ± 5%	Small wood particles mixed with resin and pressed under high heat and pressure to form a rigid board.
Bamboo	4.8	500–800	Most commonly grown in Southern Asia, very hard outer surface.
Hardwood	3	10.656	Treated panels of plywood – soluble constituents are dissolved out, proportion of lignin is increased. Produces grainless hard board with uniform strength in all direction.
Polyvinyl Chloride (PVC)	2.9	700	Thermoplastic
Polycarbonate	3.8	120	Oil based plastic, transparent, fully recyclable
Plexiglass/Perspex	5.8	1180	Polymethyl Methacrylate (PMMA), acrylic based recyclable plastic

**Table 2:** Gas Gun Material Configuration

**Material**	**Number of Layers**	**Total Thickness (mm)**
3.7 mm Strawboard	10	37.4
3.8 mm Polycarbonate	6	22.8
2.9 mm White Vinyl	6	17.1
5.8 mm Flexiglass	4	23.2
3.3 mm Hardboard	10	33
18 mm MDF	3	54
9.2 mm Chipboard	5	46
6 mm MDF	7	42
6 mm MDF	5	30
9 mm Plywood	5	45
4.8 mm Bamboo	9	43.2
4 mm MDF	10	40
5 mm Fibreboard Underlay	10	50
5 mm Fibreboard Underlay	20	100
5 mm Fibreboard Underlay	30	150

Strawboard was supplied of dimensions 1000 mm by 800 mm by 3.7 mm and was used as the baseline, while the remaining materials were identified as suitable replacements for strawboard due to their low cost, comparable dimensions, ready availability, and their conformity to relevant test standards (Table 3).

**Table 3:** Fragmenting Device Material Configuration

**Target Material**	**Total No. of Sheets**	**Total Thickness**
5 mm Fibreboard	60	300
6 mm MDF	24	144
18 mm MDF	5	90
3.74 mm Strawboard	40	149.6

### Experimental setup – Gas gun

A 22 mm bore Explosive Low Velocity Impact System (ELVIS) gas gun was used to deliver the projectile to the target material. These experiments were designed to investigate the energy absorption of these materials in a lower velocity regime, assessing whether material performance is strain-rate dependent i.e. performance changes with the impact velocity of the projectile. As these experiments were performed in the gas gun it allowed the study to be conducted in a laboratory setting as opposed to an explosive range, providing greater environmental control at lower risk and cost. The use of a gas gun also allowed a rapid and repeatable method of accelerating the ball bearing to the required velocity.

**Fig. 2:**
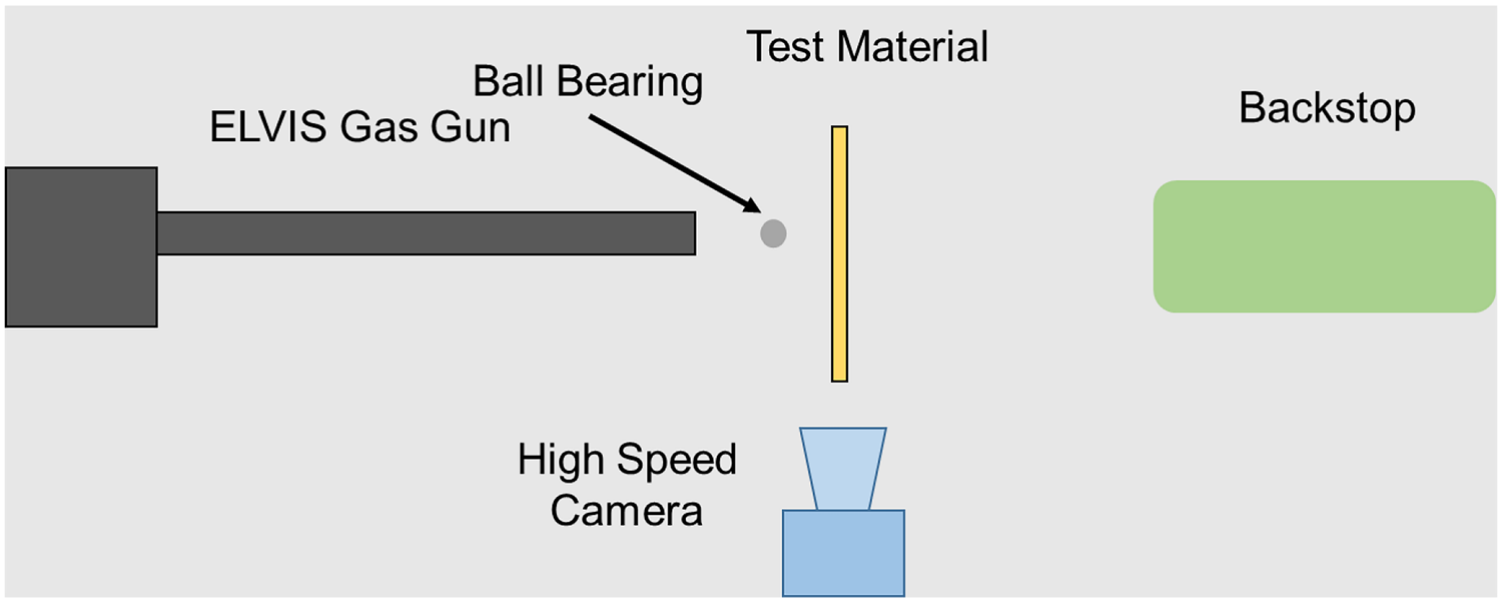
Diagram of Experimental Set Up (Not to Scale) [18]

A diagram of the experimental set up is at Fig. 2, Helium pressures of 45 bar achieved average muzzle velocities of 500 ± 50 ms^−1^ measured with light gates. The use of Helium as a driving gas delivers higher projectile velocity for the same input pressure compared to air [35]. The high-speed camera used to measure the fragment velocity pre and post perforation of the target was a Phantom V12/12 high speed camera at 40,000 fps. The velocities were then measured using Phantom Camera Control (PCC) software v2.8. Following perforation of the target (300 mm × 300 mm) the fragments were captured in a rag filled backstop which is integral to ELVIS. The fragments were not extracted for any further analysis in this case, but this soft capture mechanism prevents further damage occurring to the fragments after passing through the target, which allows for examination of the fragment size and shape distribution.

### Projectile configuration

During gas gun (ballistic) experiments, a spherical 6 mm chromed steel ball bearing of mass 0.8g was procured for use as an indicative fragment. Due to the bore diameter of the gas gun measuring 22 mm, the ball bearing was placed within a 3D printed Acrylonitrile Butadiene Styrene (ABS) plastic sabot and secured using a small quantity of plasticine. The sabot provides obturation which enables the propellant gases to expand behind the sabot, pushing the projectile out of the barrel [36]. Sabots also impart stability in flight for projectiles small than barrel inner diameter as it prevents lateral deviation to the direction travel, or tumbling on barrel exit. The sabot is then separated from the projectile at the muzzle by the sabot splitter, to prevent the sabot petals from impacting the target material and producing anomalous results [37]. The use of 3D printed designs to cradle the projectile allows for customisation of the sabot to fit a wide range of projectile and barrel diameters.

**Fig. 3:**
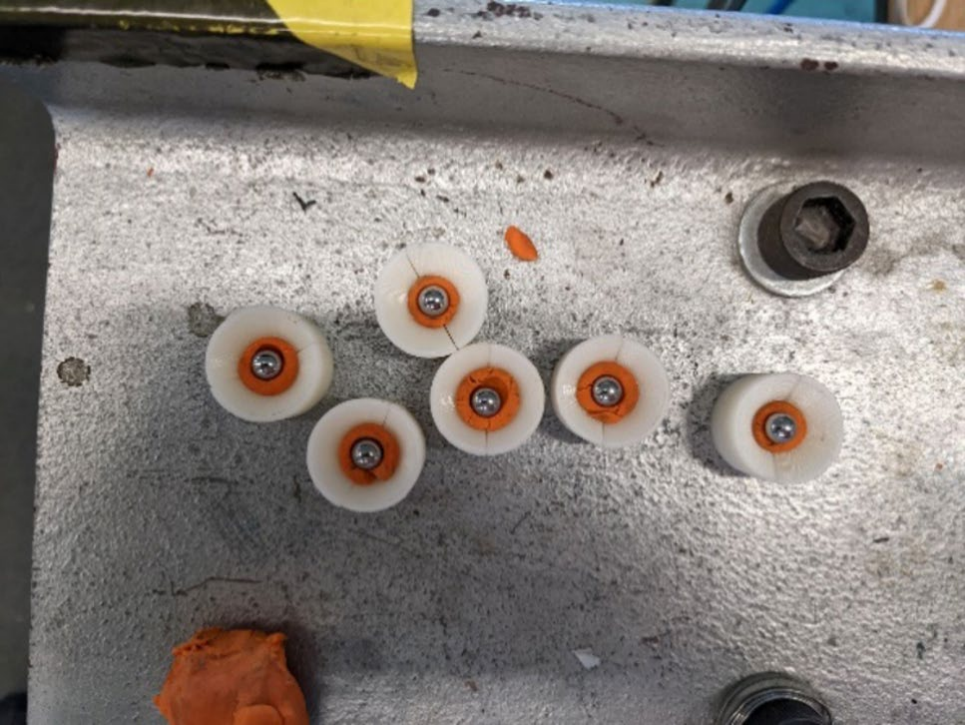
Gas gun 6 mm ball bearing projectile and 22 mm sabot set-up

The complete fragment and sabot weighed 4.15g and can be seen in Fig. 3. For the explosive test series, 6 mm steel ball bearings were used as a harder projectile capable of withstanding the blast effects generated by the explosive without deformation due to heat of explosion.

### Experimental setup – Fragmenting device

**Fig. 4:**
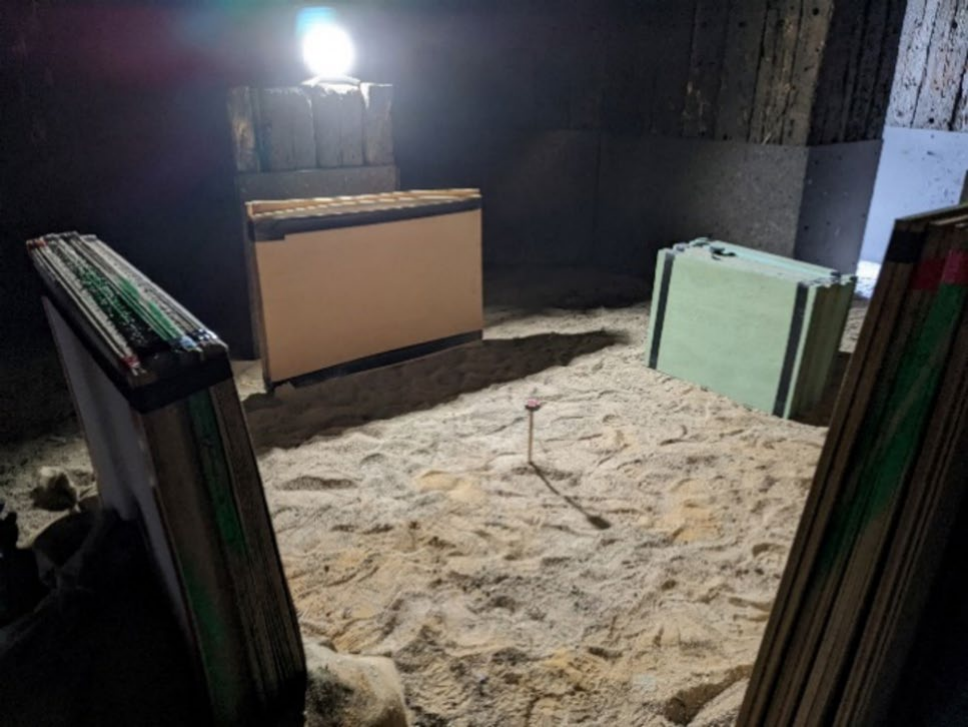
Experimental Set Up - Explosive Testing

To assess the performance of the target materials, an explosive trial was performed. Prior to detonation, target materials were layered to provide varying thicknesses and positioned to align with one of the four sides of the explosive charge as shown in Fig. 4. To maximise efficiency four materials were tested simultaneously which also assisted in reducing errors induced from excessive material set ups. They were located 1.2m from the charge on all sides. The charge itself was elevated on a pylon to ensure that a full spherical burst was achieved, because the shape of the blast wave propelling the fragments is influenced by the shape of the explosive charge, and proximity to the ground or other rigid reflective structures. A charge placed directly on the ground will form a hemispherical blast wave, with multiple subsequent ground reflection waves and Mach stem that will influence the fragmentation behaviour [38]. Positioning the charge above the ground removes the influence of ground reflections on the projectile behaviour.

For each firing, the number and position of the fragments that hit the MDF panels were recorded and tabulated using cartesian coordinates starting from the centre of the panel. The key parameter of interest in this study was depth of penetration, measured by visual inspection of the number of layers of target material the projectiles penetrated. This is indicated by the layer number at which the individual ball bearing is arrested within the target e.g. layer 7, multiplied by the layer thickness for each set up to give penetration distance.

### Fragmentation charge design

Three differing configurations of ball bearing arrangement were constructed and described in Table 4. A 65g cube of Semtex 1a was then measured and used to provide a velocity of detonation of 7000 m/s [39]. The ball bearings were secured to four outward facing sides and a standard blasting cap was used to centrally initiate the charge from above. This method ensures controlled, repeatable fragmentation set ups that are symmetrical on all sides of the charge, removing fragmentation installation pattern as a variable.

**Table 4:** Explosive Charge Fragmentation Configuration

**Test Number**	**Ball Bearing Dia. (mm)**	**Number Used**	**Rows**	**Secured to No. Faces**
1	6	24	4	4
2	6	24	4	4
3	6	24	4	4
4	5	35	5	4
5	5	35	5	4
6	5	18	3	4

## Results and discussion

### Gas gun – Average depth of penetration

**Fig. 5:**
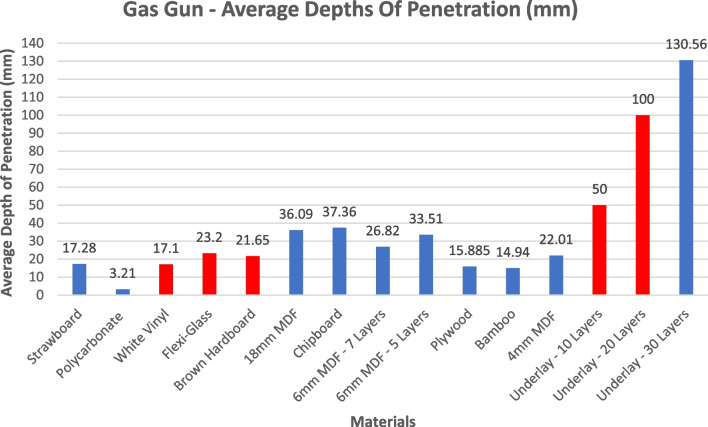
Average Gas Gun Depths of Penetration (Red Highlighting Perforation)

Figure 5 shows the measured depths of penetration achieved during the laboratory experiments, with strawboard as the control material yielding an average depth of penetration of 17.28mm. In all cases (n = 4) the shots against PVC, Plexiglass, Hardboard and both 10 and 20 layers of underlay perforated the material as indicated by the red bars. 3 materials demonstrated shallower depths of penetration (DOP) than strawboard, 5 materials yielded DOPs up to twice that of strawboard, and one material (30 layers fibreboard underlay) captured fragments at 5–6 times the depth of strawboard. Standard deviations (where applicable) of these results are shown in Table 5.

**Table 5:** DoP Standard Deviations

**Material**	**Mean DoP (mm)**	**Standard Deviation**	**St Dev as % of Mean**
Strawboard	17.28	5.818	0.337
Polycarbonate	3.21	1.853	0.577
White Vinyl	17.1	0*	0*
Flexi Glass	23.2	0*	0*
Brown Hardboard	21.65	16.051	0.741
18 mm MDF	36.09	0.969	0.027
Chipboard	37.36	12.218	0.327
6 mm MDF – 7 Layers	26.82	6.805	0.254
6 mm MDF – 5 Layers	33.51	0*	0*
Plywood	15.885	6.979	0.439
Bamboo	14.94	0.03	0.002
4 mm MDF	22.01	5.769	0.262
Underlay – 10 Layers	50	0*	0*
Underlay – 20 Layers	100	0*	0*
Underlay – 30 Layers	130.56	33.665	0.258

*Standard deviation not calculable due to limited data sets

Of the wood samples placed under test, Plywood, Bamboo, and MDF (at all thicknesses) performed most like Strawboard when considering depth of penetration as the key metric. Whilst 4 mm MDF provided closer results to Strawboard when examining depth of penetration, the 18 mm thick sample provided the most consistent results by comparison as shown in Table 5. The thickness of the 18 mm sample was originally thought to have influenced the result by increasing the areal density, however, McMahon has reported that once a material has been calibrated the thickness does not matter [29] so long as the density per unit volume remains the same. There is no discernible correlation between the number of layers (of any thickness) in a stack of MDF, or the total thickness of the stack, and the average depth of penetration, indicating that a layered structure does not have a significant impact on system behaviour.

Due to the construction of chipboard containing many sporadic sized pieces, impact with the projectile showed significant material overmatch with the material exhibiting signs of brittle failure and producing excessive amounts of fragmentation which varied in quantity dependant on the area hit. By comparison, Plywood was shown to be in excess of the hardness required producing excessive splintering and inconsistent indentation which is thought to be due to the inconsistencies in manufacture. Plywood and Hardboard produced the most splintering which has the ability to cause damage to instrumentation and interfere with HSV footage leading to inaccurate results. For the reasons listed above it was determined that Chipboard, Hardboard and Plywood are not suitable alternatives to Strawboard.

**Fig. 6:**
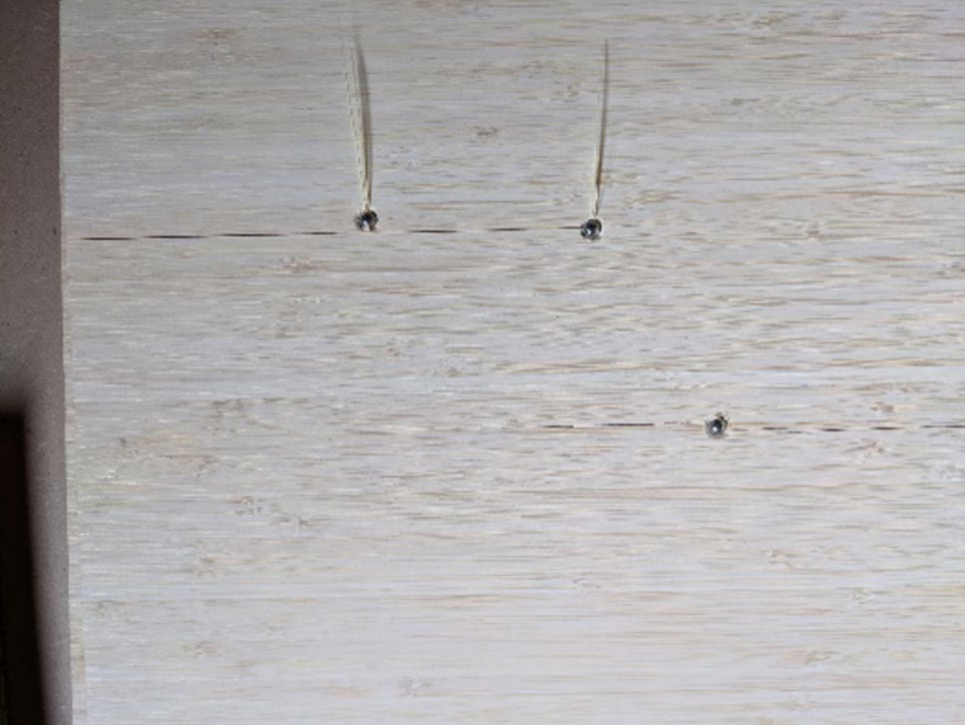
6 mm Projectile Arrest in Bamboo

Bamboo exhibited interesting results, with the front face of the material being slightly damaged by the plastic debris generated by the sabot dispersion. The ability to examine front face impact is critical to understanding how the projectile has fragmented, if any of those fragments remained lethal and consider any fragmentation interaction post blast thereby interfering with overall projectile trajectory and therefore depth of penetration. Whilst this was present, quantity was not sufficient to deter from visual inspection and both projectile impact location and trajectory was clearly visible throughout the material. Upon further inspection it was shown that all three projectiles fired into the materials had not perforated the back face and arrested without material failure – Fig. 6. Of all materials tested bamboo was the most consistent when examining depth of penetration, more so than strawboard, possibly as a result of the less variable material matrix compared to materials manufactured in the same way as strawboard. This was consistent with work previously reporting the same inconsistencies in both gas gun (ballistic) and explosive testing configurations [18]. However, when applying the guidelines detailed within NATO ARSP-03 [28] Bamboo is found not to be viable due to cost and issues found within the supply chain thereby limiting availability within the UK. Although not a viable option for the UK, the guidelines state that availability should be considered by region. It is therefore proposed that as supply is plentiful within the Asia–Pacific regions, this material be further explored to full analyse is potential as a witness material used within the Southern Hemisphere.

**Fig. 7:**
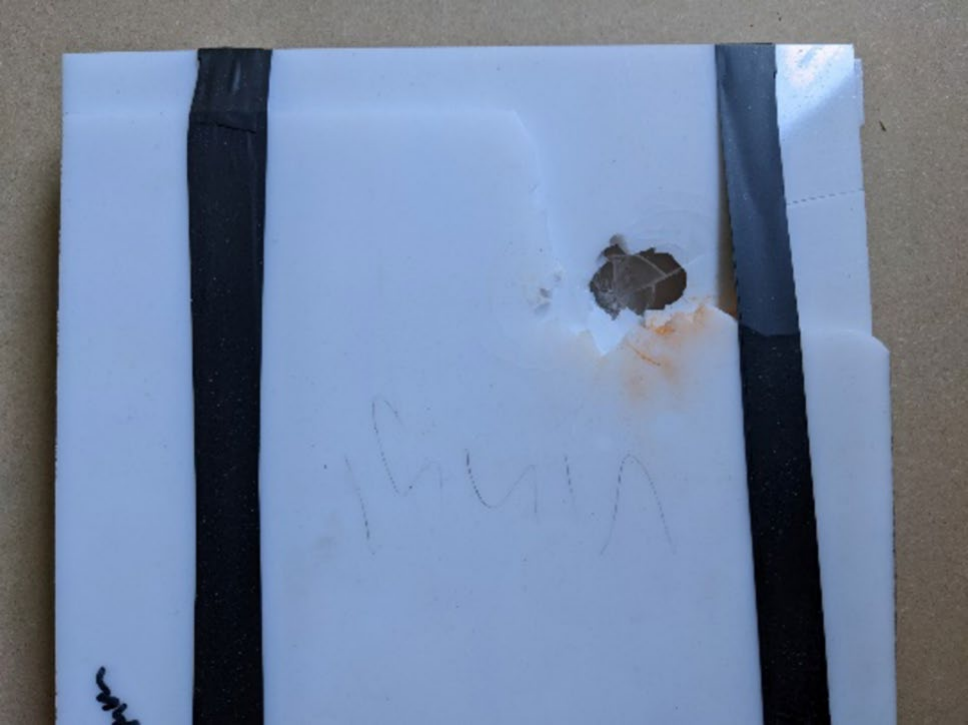
PVC post impact damage

The plastic materials placed under test performed as predicted. The PVC material shattered on impact (Fig. 7) whilst the Perspex was of sufficient strength to capture the projectile within the first layer of material. The polycarbonate was able to withstand penetration and small indentations were found to be present on the front face of the material. Previous works have suggested that the inability for the projectile to penetrate the material is due to insufficient Kinetic Energy Density values being present to overmatch the material strength [6] resulting in the polycarbonate not being adequate to measure projectile depth of penetration.

To further explore the phenomena shown by fibreboard underlay identified in previous works [18], increased quantities were layered to analyse the materials ability to withstand projectile impact. Additional layers are required because underlay has a much lower volumetric density and therefore requires greater thickness to arrest projectiles compared to other materials studied in this work. The layering of both 10 and 20 sheets of underlay resulted in full perforation of the ball bearing with significant residual velocity witnessed. To further explore this, 30 layers of underlay were placed under test (n = 3) resulting in 1 perforation and 2 penetration events. This is contrary to previous works in which underlay had been experimentally proven to be a viable economical alternative when exposed to Less Lethal Projectiles (LLP) [18]. Perforation in this case could be attributed the velocities at which the projectile was expelled from the Gas Gun. 500 ms^−1^ was used in this study whereas previous works show that ~ 260ms^−1^ exhibited results that would be preferable for witness capture material. This indicates that researchers should consider what elements of fragmentation/projectile performance they are interested in when selecting the witness capture material, and have an idea of the expected impact velocities. If pattern spread is the only variable of interest, underlay would be suitable, but not if depth of penetration also needs to be recorded.

**Fig. 8:**
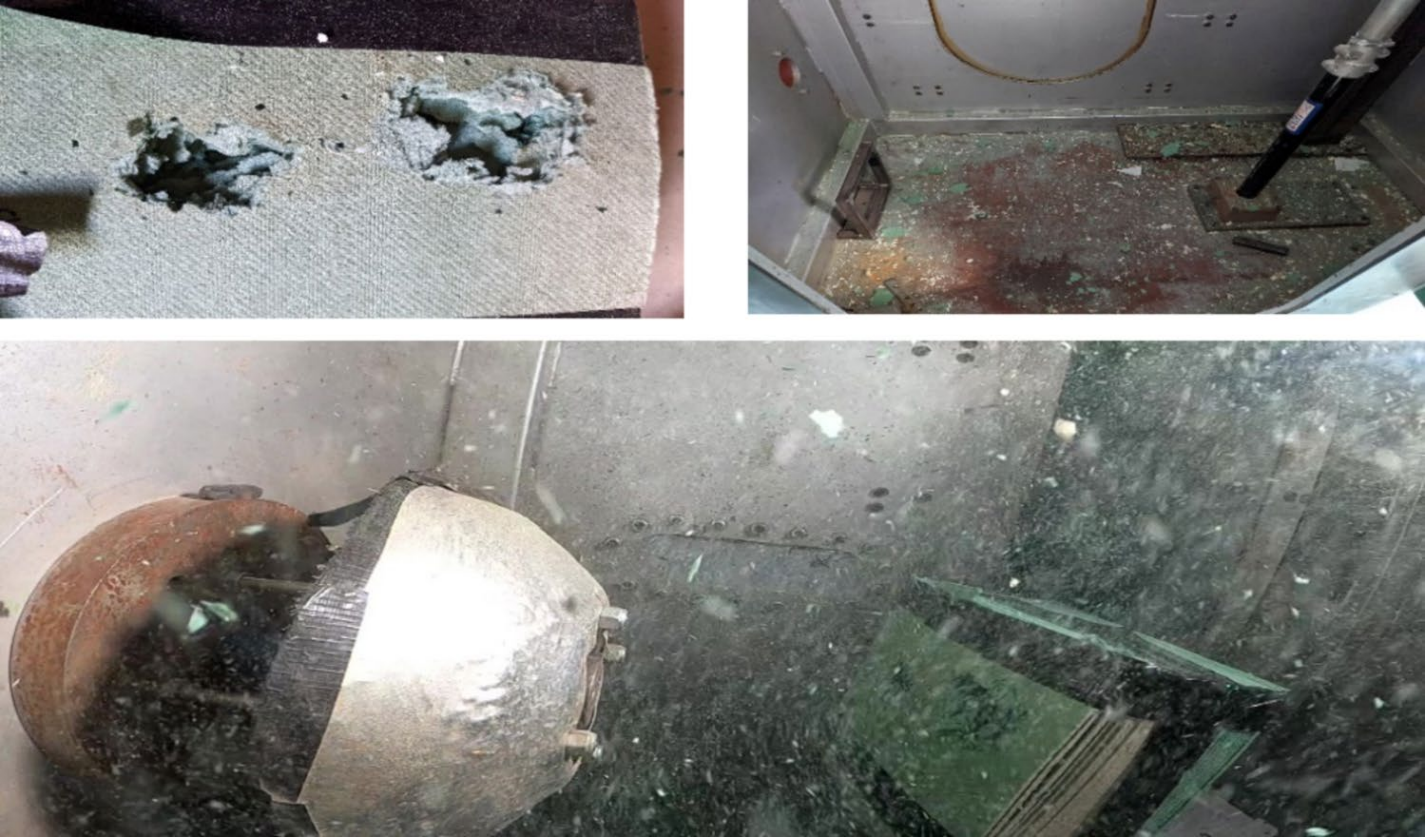
Top Left: Underlay material sample post projectile impact. Top Right: Underlay debris within target chamber. Bottom Centre: Underlay Debris post projectile impact

Although perforation was apparent in this study, assessment against NATO ARSP-03 shows beneficial characteristics when availability, flammability and recyclability are concerned [28]. However, it was found that during testing, debris caused by impact to the front and perforation of the rear faces (Fig. 8) increased times between shots and therefore required additional time to be scheduled to complete the experiments, reducing the practicality of fibreboard as an alternative. This is of concern due to the cost implications when considering the reduced time frames and increased cost pressures of current research programmes.

Post-trial analysis revealed that the most advantageous materials to provide further experimental analysis on were, Strawboard, 6 mm MDF, 18 mm MDF and 5 mm Underlay. These were down-selected due to their material performance and their ability to align with NATO ASRP-03 requirements for witness capture materials [28].

### Explosive testing

**Fig. 9:**
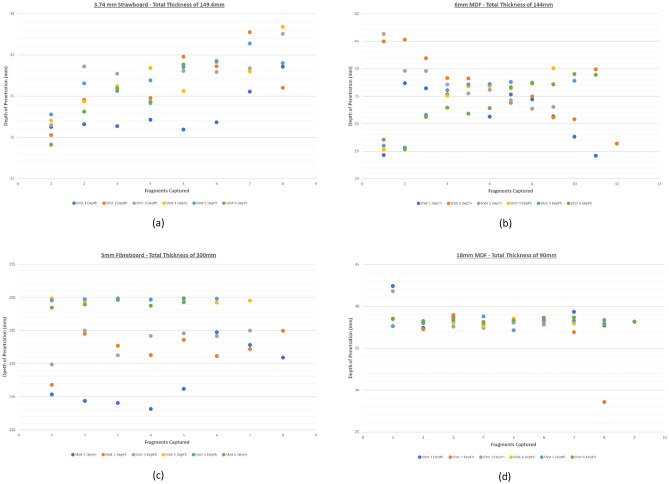
Depth of penetration (y axis) plotted against the quantity of fragments (x axis) captured during the explosive testing regime.  Top Left: 3.74mm Strawboard (149.6 mm total), Top Right: 6mm MDF (144 mm total), Bottom Left: 5mm Fibreboard (300 mm total), Bottom Right: 18 mm MDF (90 mm Total)

Figure 9 shows the results from the 3 alternative materials taken forward for explosive testing against both the 6 mm and 5 mm ball bearings. The two differing sizes were used to examine differences in velocity and/or interaction post detonation. Strawboard demonstrates inconsistencies in depth of penetration that were unexpected for a calibrated material. Fibreboard Underlay is more consistent, albeit with a far greater depth of penetration at 100mm. This is attributed to the lower material density compared to strawboard. It was also the only material to show a difference in depth of penetration between both 6 mm and 5 mm ball bearings. This is a direct resultant of a lower yield material being impacted by the 5 mm ball bearing at increased velocity when directly compared to the 6 mm ball bearing. This is resulted in greater depth of penetration.

6 mm MDF shows similar depth and variation of penetration to strawboard, whereas 18 mm MDF is far more consistent with the vast majority of fragments captured between 37 and 39 mm depth of penetration. All materials had a shot pattern spread of approximately 15-25 mm.

Exposing the alternative materials to a 65g charge of plastic explosive ‘Semtex’ 1a (n = 6) saw contradictions to the results seen under laboratory Gas Gun (ballistic) conditions. Of particular interest was the underlay, which showed penetration of the fragmentation at depths of 130–145 mm in comparison to the 30–45 mm depth of Strawboard and MDF during the first 3 shots which included being exposed to a total of 24 6 mm ball bearings per material. To further explore the possibility of interaction between the ball bearings impacting the results seen under explosive conditions, 6 mm diameter ball bearings were replaced with hardened 5 mm ball bearings. The reduction in diameter produced similar levels of fragmentation trajectory but deeper penetration to all materials which was more noticeable within the underlay (increase of 4 mm +/- 1 mm).

Whilst the marginal change in projectile diameter was advantageous, it indicated that a change in size, density and velocity of the fragment was not sufficient to influence results on the Strawboard and MDF materials under test. Fibreboard Underlay however, possesses a greater sensitivity to impact speed with a consistent increase in depth of penetration, indicating that it provides a more accurate measure of penetration depth relative to impact speed. The harder MDFs lack this level of measurement resolution.

Repeatability is a key factor when considering an economical alternative and each material under test was shown to withstand high speed impacts from 40–60 fragments at once. Whilst this is important, the ability to further extrapolate data from the witness panels post detonation is extremely desirable and all materials under test were shown to retain their structural integrity upon removal from the test arena.

Underlay was again of interest here, retaining its shape and structure post detonation which is not in keeping with the results shown during the gas gun (ballistic) experimental campaign. The possibility to analyse results, mark them as acknowledged and re-use the same material is a key finding as economical alternatives not only need to consider time and cost but also environmental impact during disposal. Repeatability is linked to the structural integrity of the material – whilst consistent behaviour between different targets can be seen, each individual target can be used far fewer times than the other alternatives and strawboard.

An explanation for the differences in underlay behaviour is that slower impact speeds as seen in the gas gun (ballistic) experiments cause the ball bearings to dump all the energy into the underlay, tearing it apart. The higher explosive speeds mean the projectiles perforate and therefore energy is not transferred and absorbed by the underlay, so it retains its structure. This is a phenomenon seen in wound ballistics with very different damage patterns seen by the projectiles that come to a rest within the body compared to those that exit the body [5].

In order to analyse the performance of the projectile under test, the witness capture material must not influence the trajectory of the projectile or cause deformation. For this study, all projectiles were easily recoverable and retained their shape allowing them to undergo post impact analysis/ hardness testing if required. This trait is clearly linked to the density of the material being used as a witness screen and is therefore recorded within NATO ARSP-03 [28]. 6 mm MDF, 18 mm MDF and 5 mm Fibreboard performed most advantageously when compared to the baseline comparator strawboard during both explosive and ballistic experiments. All materials under test exhibited the same failure modes, regardless of test environment or setup therefore allowing for the materials in question to be interchangeable between both explosive and ballistic set ups dependant on requirements. The data presented has shown that regardless of requirement, MDF is the highest performing material and should be further quantified to increase awareness of performance in a broader range of settings.

To further analyse the potential cost savings, a comparison to the strawboard test series mentioned during the introduction was undertaken. A total of 1800 1m × 0.8m strawboard sheets were required at a cost of £12 per sheet, totalling £21,000. By comparison, a 2.44 × 1.22m MDF sheet costs £14.45 from a UK timber merchant wholesale, however, this would be segregated into thirds to match the strawboard sizes. The total per MDF sheet therefore would equate to £4.82. It should be noted that greater economic return can be had if bulk orders are placed, further reducing the cost to £4.55 per sheet when quantities greater than 150 sheets are purchased together. For the remainder of this comparison, the worst case £4.82 was used.^2^

6 mm MDF sheets would enable the user to use less panels during the test series when based on the experiments undertaken within this study. By analysis, 6 sheets of 6 mm MDF would perform similarly to 10 sheets of Strawboard. When compared to the strawboard example listed above, this would equate to a total cost of £5205.60 for 1080 MDF panels, clearly demonstrating the economic value that MDF presents in comparison to Strawboard.

2.44 m × 1.22 m sheets of 18 mm MDF are priced at around £35.84 incl. VAT from the same wholesaler, and would require 2 sheets per 10 strawboards. Using the same logic as above for the 6 mm MDF area calculations, this produces a total cost of £12,902.40, just under half that of the strawboard. Underlay costs £19.99 per pack of 25 sheets 590 mm × 850 mm × 4 mm. As they are approximately the same width but half the height of strawboard 3600 sheets would be required to replicate the arena trial, totalling £2878.56.

The manual handling burden is much higher for the 18 mm MDF as the sheets are far thicker and heavier, needing 2 persons to manoeuvre whereas strawboard only requires one person. 6 mm MDF requires fewer sheets which are much lighter, and whilst underlay requires more sheets than strawboard it is much lighter and more flexible therefore easier to manage, thus both these materials have a reduced manual handling burden compared to strawboard. All of the alternatives are far more readily available than strawboard, as there are numerous suppliers within and available to the UK.

## Conclusions

This paper has explored the economic viability of Strawboard, 6 mm MDF, 18 mm MDF and 5 mm Underlay as a witness capture material to be used within both research fields exploring ammunition lethality as well as reconstruction of crime applications where blast and fragmentation have been used against a static target. The outcomes of both Gas Gun (ballistic) and Explosive testing have shown that 6 mm MDF panels performed the highest when depth of penetration is the key metric and when scoring against the NATO guidance document relating to witness capture material.

6 mm MDF has been shown to be readily available on the open market, easy to handle and able to be used repeatedly to reduce environmental impact. 18 mm would be viable but greater manual handling burden and more expensive, whilst underlay shows promise in less lethal kinetic impact applications but is messier to use and does retain structural integrity for multiple shots or post-experimental examination.

Further work on 6 mm MDF survivability against a range of charge and projectile geometries should be explored to fully analyse versatility of the material.

## Key points

- 10 commercially available target materials have been exposed to ballistic and explosive assessment for viability as an economic replacement for Strawboard as a witness capture material.
- MDF has shown increasing promise as a suitable alternative to Strawboard.
- Less Lethal Kinetic Impact scenarios are considered for materials that exhibited signs of failure during ballistic and explosive trials.
- Recommendations to improve environmental and economic practise with commercially available materials are made to better align research programmes with wider environmental initiatives.

## Data Availability

New data has been created and reported as part of this study. Data is available to share on request.

## Footnotes

1 Cost per sheet at time of purchase (2022).

2 All costs correct at the time of writing.
